# Psychometric Properties of the Self-Concealment Scale in Spanish Adolescents: Adaptation and Validation for Eating Disorder Risk Assessment

**DOI:** 10.62641/aep.v53i3.1857

**Published:** 2025-05-05

**Authors:** Ana González-Menéndez, Víctor González-Suárez, María José Medrano, Maria-João Alvarez, Álvaro Postigo

**Affiliations:** ^1^Department of Psychology, University of Oviedo, 33003 Oviedo, Spain; ^2^Clínica IPA, 33002 Oviedo, Spain; ^3^Equipo de Orientación Educativa de Nervión, Junta de Andalucía, 41005 Sevilla, Spain; ^4^CICPSI, Faculdade de Psicologia, Universidade de Lisboa, 1649-013 Lisbon, Portugal

**Keywords:** self-concealment, body image inflexibility, eating disorders, adolescents

## Abstract

**Background:**

Recent studies on transdiagnostic processes consider eating disorder (ED) examples of psychological inflexibility. To date, the instrument most widely used to evaluate self-concealment is the Self-Concealment Scale (SCS), although there is as yet no Spanish adaptation of the instrument. The objective of this study was to adapt and study evidence of validity of the SCS to the adolescent population in Spain.

**Method:**

A sample of 230 Spanish adolescents aged 13 to 19 years (Mean (M) = 15.52; Standard deviation (SD) = 1.13) was used to study the psychometric properties of the SCS. The discriminative capacity of the items was analyzed, their unidimensional factorial structure was confirmed, the reliability of the scores was studied, and evidence of validity in relation to other clinical variables was examined.

**Results:**

As in the original scale, confirmatory factor analysis showed adequate fit of the 10-item one-dimensional model (Standardized Root Mean Square Residual (SRMSR) = 0.05; comparative fit index (CFI) = 0.90). Body image inflexibility, and to a lesser extent, self-concealment, explained 52.2% of the variance in risk of ED.

**Conclusions:**

It was concluded that the Spanish version of the SCS has adequate psychometric properties and may be a useful tool in evaluating risk of ED in adolescents.

## Introduction

Most people have some secret they try to hide [1], negative information they do not want others to know about. Such concealment includes a certain emotional burden, as keeping back information involves constantly weighing whether it is better to protect oneself by hiding secrets or, on the contrary, opening up and revealing them [2]. The self-concealment construct described in 1990 by Larson and Chastain [3] synthesizes this dynamic. Self-concealment (S-C) refers to the psychological tendency to hide personal, distressing, and potentially embarrassing information [3, 4]. The term also describes actively and consciously hiding information on negative thoughts, emotions, open behavior, or experiences. The purpose is to maintain a positive image of oneself and keep others from feeling deceived or upset [1]. Although Larson and Chastain [3] originally suggested that it is the most traumatic experiences that are most frequently concealed, more recent studies also link S-C to personal inadequacy, rumination, guilt, and worry [1, 5].

Self-concealment has been studied in the context of several different disorders and maladaptive behaviors. It has been related to the severity of depression [6] and anxiety [7, 8], as well as suicidality [9], suicidal behavior [10], and non-suicidal self-injury [11, 12]. S-C has also been linked with insecure attachment [1], substance use in female adolescents [13], and maladaptive perfectionism [14]. Therefore, there is robust empirical evidence that self-concealment is linked negatively to mental health [15].

Self-concealment has become an entire line of research in the framework of eating disorder (ED), especially anorexia and bulimia nervosa. Many studies have suggested that persons with ED tend to retain and distort information on their eating and purging habits and their negative body image [16], and that hiding or denying these points can predict the presence and severity of non-suicidal self-harm and increase the risk of suicide in those diagnosed with ED [17].

Maladaptive eating patterns seem to be linked to the suppression of distressing emotions and thoughts, and therefore, as dysfunctional ways of regulating emotions. A study of interest in this respect demonstrated that women with an ED who assumed the role of someone who did not have an ED (suppressors) experienced more intrusive thoughts than those who answered questions on body image, their weight, and dietary habits truthfully [18]. EDs may therefore be considered examples of inability to flexibly manage emotions and negative thoughts related to food, or more specifically, as forms of inflexible body image [19, 20]. S-C has been found to be inversely proportional to psychological flexibility [21], to cognitive and emotional aspects typical of EDs [22, 23, 24], and, in general, to behavior characteristic of eating problems [25]. Flexible body image is also a relatively new construct referring to the extent to which a person is able to openly experience body dissatisfaction and the distressing thoughts derived from it without acting consequently, and making no effort to avoid or change them [20]. It is clarifying to observe the way in which different behaviors having to do with self-induced starvation and purging habits express discomfort, disgust, or nonconformity with oneself [21, 26]. Studies on emotion regulation strategies in ED patients also show that symptoms such as restriction or purging function as maladaptive efforts to escape from negatively judged emotional states [27]. While S-C reflects rigid efforts to suppress and avoid thoughts, feelings, and unpleasant events, flexibility with body image reflects efforts at psychological acceptance and openness to distressing events, and particularly acceptance of discomfort and dissatisfaction with weight and body image [21].

The Self-Concealment Scale (SCS), designed by Larson and Chastain [3] in 1990, is one of the most widely used instruments for evaluating self-concealment. It is a 10-item questionnaire answered on a five-point Likert-type scale referring to three different aspects: tendency to keep things to oneself, possession of a personal secret not shared with anyone else, and fear of revealing such information [3]. The total score is the sum of the ten items, such that the higher the score, the greater self-concealment is. First evidence showed that it was a one-dimensional reliable instrument with test-retest reliability (at four weeks) and interitem reliability coefficients of 0.81 and 0.83, respectively [3]. A review of 99 studies analyzing the scale’s Cronbach’s alpha reported a mean coefficient of 0.87 and confirmed that S-C differs empirically and conceptually from self-revelation [4].

Evidence of the validity of the SCS has been proven in different cultural contexts, finding that the scale has a certain transcultural applicability. The original English-language scale has been translated into many languages, including German [28], Chinese [29], French [30], Portuguese [31], and Japanese [32].

The objective of this study was to begin the process of adaptation to Spanish and validation of the SCS for Spanish adolescents. Evidence of validity was studied as a function of the internal structure of the Spanish version of the SCS, reliability of its scores, and evidence of validity of the Spanish version of the SCS as related to other variables such as body image inflexibility (BII) and eating disorder (ED) risk behaviors.

## Method

### Participants

An incidental sample of 230 adolescents in the third and fourth years of middle school and first and second years of high school. The students were recruited through convenience sampling through several schools. All of them participated voluntarily in the study and did not receive any incentive for their participation. The mean age of the participants was 15.52 years (Standard deviation (SD) = 1.13; range: 13–19 years). Boys made up 54.8% of the sample (n = 126), while 45.2% were girls (n = 104), 40.4% were in middle school (n = 93), and 59.6% were in high school (n = 137). The inclusion criteria were between 13 and 19 years old. Students over the age of 18 could exercise a free and informed decision to participate in the study. Students older than 19 years were not included to reduce sociodemographic heterogeneity. Students with intellectual disability were excluded.

### Instruments

Self-Concealment Scale (SCS; [3]). This is a self-report inventory evaluating the tendency to conceal personal information that may be stressful or negative. The questionnaire contains ten items on a Likert-type scale from 1 (strongly disagree) to 5 (strongly agree), in such a way that the higher the score, the greater the self-concealment. The total score is calculated as the sum of the scores on all ten items, with all items being positively worded [33]. The scale’s reliability was shown by a Cronbach’s alpha of 0.81 [3]. The first step in adapting the SCS was its translation following international standards for test translation and validation [34, 35]. For back-translation, we first required the support of three bilingual natives. One of them translated the test into Spanish and the other translated it back into English. Then a third person compared that English version with the original. Finally, three experts analyzed the wording of each of the translated items, and by consensus, reworded some of them.

Body Image-Acceptance and Action Questionnaire (BI-AAQ-12; [21]). The BI-AAQ measures body image psychological flexibility/inflexibility. This instrument evaluates flexible forms of responding to negative thoughts related to body image and physical appearance. It has 12 items with seven answer choices from 1 (strongly disagree) to 7 (strongly agree). Scores go from 12 to 84 and higher scores imply stronger inflexibility. The scale has a reliability coefficient of 0.92 [21, 36].

Spanish Version of the Eating Disorders Examination Questionnaire (S-EDE-Q;[37]). The Spanish adaptation by [38] was used. The questionnaire was designed to identify the presence of ED by means of 36 generic items and two more directed at women (regarding menstruation). In addition to a total scale, it includes a specific assessment of attitudes about patterns of restraint (R), eating concern (EC), shape concern (SC) and weight concern (WC), as well as problematic behavior.

The response format of those items is a 7-point Likert-type scale (0: never, 6: everyday). The global score is the average of the four subscale scores. The frequency of key ED eating and compensatory behaviors is assessed in terms of the average number of weekly episodes occurring for the past four weeks. The Cronbach’s Alpha for the subscales is 0.93 for shape concern, 0.86 for restraint, 0.75 for eating concern, and 0.74 for weight concern [38].

### Procedure

All the participating adolescents, as well as their parents or legal guardians, were first informed of the purpose of the study and then agreed to collaborate in it by signed consent. Subsequently, the researchers explained to the students the purpose of the study, the instructions for answering the battery of tests, and they were asked to raise their hands when they had any questions, so that they could be clarified, and they could continue answering. All of them were ensured that their answers would be anonymous.

The instruments were administered by a single expert evaluator in the following order: BI-AAQ-12, S-EDE-Q and SCS, and took from 20 to 25 minutes. Data collection was conducted in November and December 2022.

This study was conducted in line with the Declaration of Helsinki. The University of Oviedo Ethics Committee previously approved this research. In addition to obtaining permission slips from the schools where the instruments were administered and informed consent forms signed by parents or guardians, the study adhered to the University of Oviedo’s code of good practices for data protection, which was approved by the Faculty of Psychology.

### Data Analysis

First, the descriptive statistics (mean, standard deviation, asymmetry and kurtosis) of the ten items, and then the item discrimination indices (corrected item-test correlation) were studied. The Shapiro-Wilk method was used to test the assumption of normality. Restraint, eating concern, shape concern and weight concern on the eating behavior questionnaire did not meet the normality assumption (*p* < 0.05). These variables will be presented with the median and with the 25th and 75th percentile.

A confirmatory factor analysis (CFA) was performed to analyze the SCS factor structure, and with it, evidence of validity based on internal structure, thereby testing the one-dimensional structure found in previous studies [2]. As the items were ordinal, maximum likelihood estimation was used for analysis with two fit indicators: two absolute indices (Standardized Root Mean Square Residual, SRMSR; and Root Mean Square Error of Approximation, RMSEA) and two related indices (comparative fit index, CFI; and Tucker-Lewis index, TLI). Fit is considered good when SRMSR and RMSEA values are 0.08 or lower and excellent when they are 0.05 or lower, and TLI and CFI values are 0.90 or higher. Reliability of the scores was studied with the Cronbach’s alpha.

For validity evidence in relationship with other variables, several *U* Mann-Whitney were done by sex. Apart from this, to find convergent validity evidence, the Pearson’s correlation was used to study the relationship of the SCS to the various clinical variables in the study. Second, a linear regression analysis (stepwise) considering the scores on eating attitudes as the criterion variable, and as “predictor” variables, the measures of body image flexibility and self-concealment. Statical significance was set at *p* < 0.05 and *R*^2^ was employed to find the percentage of variance explained by the criterion variables.

Statistical analyses were conducted with Jamovi 2.3.28 (Jamovi Open Stats, Sidney, Australia) [39], which was created by Jonathon Love, Damian Dropmann, and Ravi Selker.

## Results

### Descriptive Statistics and Item Analysis

The results of the descriptive analyses are shown in Table 1. The sample was made up of 54.8% boys and 45.2% girls, with a mean age of 15.52 ± 1.13 years. All of them were in middle school Grade 3 to high school Grade 2.

**Table 1.** **Sociodemographic and clinical characteristics of the sample (N = 230)**.

Age (M, SD)		15.52	1.13
Sex (F, %)			
	Male	126	54.8
	Female	104	45.2
Academic level (F, %)			
	3° ESO	53	23
	4° ESO	40	17.4
	1° Bachiller	97	42.2
	2° Bachiller	40	17.4
SCS (M, SD)		23.48	9.61
BI-AAQ-12 (M, SD)		30.6	15.61
Restraint median (P25; P75)		0.40	(0; 1.80)
Eating concern median (P25; P75)		0.20	(0; 0.85)
Shape concern median (P25; P75)		0.75	(0.13; 1.78)
Weight concern median (P25; P75)		0.60	(0; 1.80)
Global score median (P25; P75)		0.58	(0.18; 1.58)

SCS, Self-Concealment Scale; BI-AAQ-12, Body Image-Acceptance and Action Questionnaire; M, Mean; SD, Standard deviation; P25, percentile 25; P75, percentile 75; F, frequency; ESO, Secondary Education.

The results of the clinical variables analyzed showed that mean body image flexibility (BI-AAQ-12) was 30.6 (SD = 15.61). For the non-normal continuous variables, restraint, eating concern, shape concern and weight concern on the eating behavior questionnaire, median, P25 and P75 were used. It is shown in Table 1.

First the descriptive statistics of the items are shown in Table 2. Asymmetry and kurtosis are adequate for all of them. Discrimination indices are all high, varying from 0.49 to 0.66 (Table 2). The mean participant score on the self-concealment scale was 23.48 (SD = 9.61).

**Table 2.** **Descriptive statistics of the items from the Spanish version of the Self-Concealment Scale (SCS)**.

Item	Mean	Standard deviation	Asymmetry	Kurtosis	Item-test correlation	Factor loading
1. I have an important secret that I haven’t shared with anyone.	2.52	1.57	0.46	–1.34	0.61	0.67
[Tengo un secreto importante que no he compartido con nadie]						
2. If I shared all my secrets with my friends, they’d like me less.	1.80	1.18	1.44	1.09	0.51	0.55
[Si compartiese todos mis secretos con mis amigos, podría gustarles menos]						
3. There are lots of things about me that I keep to myself.	3.03	1.53	–0.03	–1.48	0.64	0.67
[Hay muchas cosas sobre mí que me guardo para mí mismo]						
4. Some of my secrets have really tormented me.	2.29	1.51	0.69	–1.07	0.61	0.66
[Algunos de mis secretos me han atormentado realmente]						
5. When something bad happens to me, I tend to keep it to myself.	2.85	1.43	0.17	–1.28	0.56	0.59
[Cuando algo malo me pasa, tiendo a guardármelo para mí mismo]						
6. I’m often afraid I’ll reveal something I don’t want to.	2.14	1.35	0.86	–0.56	0.57	0.60
[A menudo temo que voy a revelar algo que no quiero]						
7. Telling a secret often backfires and I wish I hadn’t told it.	2.38	1.41	0.53	–1.08	0.55	0.59
[Contar un secreto a menudo me resulta contraproducente y deseo no haberlo contado]						
8. I have a secret that is so private I would lie if anybody asked me about it.	2.52	1.59	0.52	–1.32	0.66	0.73
[Tengo un secreto que es tan privado que mentiría si alguien me preguntase al respecto]						
9. My secrets are too embarrassing to share with others.	1.90	1.27	1.29	0.48	0.62	0.66
[Mis secretos son demasiado embarazosos para compartirlos con los demás]						
10. I have negative thoughts about myself that I never share with anyone.	2.18	1.43	0.88	–0.65	0.49	0.52
[Tengo pensamientos negativos sobre mí mismo que no comparto con nadie]						
Total	23.48	9.61	0.50	–0.51	-	-

### Validity Evidence Based on Internal Structure and Score Reliability

Confirmatory Factor Analysis backed the unidimensional scale, with adequate fit of the model (SRMSR = 0.05; RMSEA = 0.08; CFI = 0.90; TLI = 0.86). Factor loads on each item varied from 0.52 to 0.73 (Table 2). All items had factor loadings over 0.50, providing significant information, so that none of them was excluded from the Spanish version of the scale. Also, SCS reliability scores in this study were very high (α = 0.866). The final translated version of the SCS is shown in Table 2.

### Validity Evidence in Relationship With Other Variables

When the scores were compared by sex, and after testing equality of variances, statistically significant differences between sexes were observed in all the measures analyzed except self-concealment (Table 3). Since there were seven comparisons, the type I error was corrected with Bonferroni, and *p* < 0.007 was required to detect statistically significant differences. In all cases, girls scored significantly higher.

**Table 3.** **Differences in clinical variables based on sex**.

	Sex	Average range	*U* Mann-Whitney	*p*
Restraint	Men	98.52	4413	<0.001
	Women	136.07		
Concern about eating	Men	99.50	4536	<0.001
	Women	134.88		
Concern about body image	Men	90.40	3390	<0.001
	Women	145.90		
Concern about weight	Men	95.80	4069.500	<0.001
	Women	139.37		
Total score (S-EDE-Q)	Men	93.52	3783	<0.001
	Women	142.13		
Body image flexibility	Men	101.26	4757.500	<0.001
	Women	132.75		
Self-Concealment	Men	111.28	6062	0.664
	Women	115.07		

S-EDE-Q, Spanish Version of the Eating Disorders Examination Questionnaire.

Moreover, all the variables were positively and significantly correlated to Self-Concealment Scale (SCS). Positive correlations were observed between ED and body image inflexibility (*r* = 0.714; *p* < 0.001), and between ED and SCS (*r* = 0.302; *p* < 0.001). The correlation between self-concealment and body image inflexibility was *r* = 0.249 (*p* < 0.001). Correlation analysis of the subscales that evaluate risk of eating disorders reported positive statistically significant correlations between all the subscales and the body image inflexibility and SCS variables (Table 4).

**Table 4.** **Correlation matrix between the study variables**.

	Concern about eating	Concern about body image	Concern about weight	Total score (S-EDE-Q)	Body image flexibility	Self-Concealment
Restriction	0.628^**^	0.722^**^	0.689^**^	0.843^**^	0.514^**^	0.227^**^
Concern about eating		0.761^**^	0.732^**^	0.846^**^	0.606^**^	0.256^**^
Concern about body image			0.899^**^	0.944^**^	0.725^**^	0.325^**^
Concern about weight				0.925^**^	0.714^**^	0.271^**^
Total score (S-EDE-Q)					0.714^**^	0.302^**^
Body image flexibility						0.249^**^

^**^p < 0.001.

Finally, Table 5 presents the coefficients of the statistically significant variables for the model with the best fit. Intercept represents the expected value of the dependent variable (y) when all independent variables (x) are equal to zero. It may be observed that the image inflexibility and self-concealment variables entered in the regression equation, where image inflexibility was the most powerful predictor explaining risk of ED. The model explains 52.2% of the variance in risk of ED.

**Table 5.** **Predictor variables of the risk of eating disorders**.

	Unstandardized coefficients		Standardized coefficients	*t*	*p*	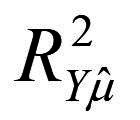	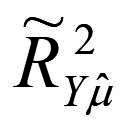
	B	Standard Error	Beta				
Intercept	–0.876	0.154		–5.687	<0.001		
Body image flexibility	0.049	0.003	0.681	14.440	<0.001	0.526	0.522
Self-Concealment	0.016	0.006	0.133	2.824	0.005		

Note. Dependent variable = Global Score on the S-EDE-Q.

## Discussion

The objective of this study was to adapt and analyze the psychometric properties of a Spanish version for adolescents of the Self-Concealment Scale (SCS). In so doing, the relationships between risk eating behavior and dysfunctional emotional regulation, such as body image inflexibility and self-concealment, were analyzed in a sample of adolescents of both sexes.

The SCS, originally designed by [3], is a one-dimensional 10-item scale which collects information on three main aspects of self-concealment: the tendency to keep things to oneself, having secret stressful personal information, and fear of revealing such information [3]. The results of the confirmatory factor analysis showed that the unidimensional model fit the data well and that the original instrument’s ten items are significantly homogeneous, and therefore, necessary to predict interference derived from hiding stressful personal information. Exploration of the SCS dimensional structure has always been a concern of research. The results presented here support previous adaptation studies [29, 31, 40] but differ from the classic study by [41]. Whereas the Exploratory Factor Analysis of that study had originally suggested that the SCS consisted of two subscales (keeping secrets and personal concealment), the data in our study support an essentially unidimensional scale. In our study, the item with the highest loading was “I have a secret that is so private that I would lie if someone asked me about it”, (0.52), so all of them were considered relevant in this adaptation of the SCS.

The results of the study also report that the adolescents in the sample did not show risk eating behavior worthy of concern. Compared to studies similar to ours in samples of young women [37, 42, 43], the adolescents evaluated here had low-risk scores. But in contrast to those studies, ours included boys as well as girls. When the general scores on the instrument that evaluated risk of ED (S-EDE-Q) were analyzed, boys were found to have scored significantly lower, confirming that our results agree with those reported in previous studies.

The same differences between sexes were observed in the rest of the variables analyzed, except for S-C. Confirmation of significantly higher scores by girls than boys on body image inflexibility, weight concern and image, agreed with previous studies [44] and emphasize the influence of social pressure on the supposedly ideal female beauty [21, 42].

However, our observations differed from those of Masuda’s group [44, 45] in that self-concealment was not associated with being female, and there was no validity evidence for this sociodemographic variable. The mean scores were very similar in both boys and girls, which would indicate that concealment could also reveal eating disorders in the subgroup of men.

In this study, robust relationships were observed between body image and restraint behaviors and eating, image and weight concerns. This is consistent with other findings which consider EDs psychological inflexibility problems or failed attempts at regulating emotions, thoughts or negative feelings [22, 23, 24].

The results of the predictor study are also revealing. The self-concealment variables, and especially, image inflexibility, explained 52% of the variance in risk eating behavior. The results of this study widen the literature on people with ED symptoms who tend to keep such problems secret to the point of denying the disorder [46] and certify that intolerance of negative thoughts and emotions about one’s own body lead to concealment, understood here as a maladaptive form of regulating negative affect. This intolerance is characteristic of the transdiagnostic process called psychological inflexibility [47]. Although many people show dissatisfaction with their image and weight, especially compared to ideal models of beauty, only a minority develop an eating disorder, which suggests that the mere presence of distressing thoughts is not inherently toxic. It is how one responds to those thoughts that generates such toxicity. Therefore, the instrument designed to evaluate avoidance behavior in eating disorders (BI-AAQ-12) does not focus on the context of body dissatisfaction, but on fusion with negative thoughts about the body and their rejection or avoidance. As pointed out by [20], it is not dissatisfaction with one’s body which triggers an ED, but whether the response to them is flexible or inflexible.

Finally, given the results of the regression analysis and the high predictive power of the BII, we can conclude that the relationships between self-concealment and risk eating behavior are at least partially explained by the process known as psychological inflexibility. The results suggest that the links between self-concealment and ED symptoms are established through shared diminished psychological flexibility traits.

In any case, and as observed in previous studies in which S-C is related to other negative health outcomes [9, 15, 48], our study highlights the importance of including strategies that facilitate disclosure and increase psychological flexibility or acceptance of aversive private content (thoughts, emotions, memories, etc.).

Some limitations of the study should be kept in mind. The sample is incidental and in a specific age range, so these results should not be generalized to clinical populations or other age ranges. Neither was the sample the ideal size for a nonclinical population, with the consequent limitations for testing the psychometric characteristics of the SCS questionnaire adequately. Therefore, longitudinal studies are necessary in future, to further clarify the causal relationships between these variables and to study the stability of the instrument scores.

In any case, the evidence presented here joins what has been found in previous studies documenting the predictive value of concealment and avoidance of distressing events in the development of many psychopathological disorders [48], and is, therefore, a first approach to the analysis of the predictive power of self-concealment and can be a starting point for adolescent intervention, following the example of the Psicología Basada en la evidencia en Contextos Educativos (Evidence-based Psychology in Educational Contexts) (PSICE) program [49].

Self-concealment, evaluated using the SCS, has a negative impact on the psychological health of Spanish adolescents and influences the development of maladaptive eating behaviors. It was therefore imperative to examine its psychometric properties and provide a valid tool favoring the development of future studies involving concealment. The SCS has been shown to be a reliable tool with validity evidence for its use in evaluating self-concealment in a Spanish adolescent population.

## Conclusions

The Spanish version of the Self-Concealment Scale (SCS) was identified to be reliable and supported by evidence of validity for assessing the tendency to conceal painful and negative information about oneself. Considering that self-concealment is high in adolescents it is pivotal to improve the assessment, intervention, and research on this construct in adolescents to minimize its potential harm.

## Availability of Data and Materials

The datasets generated and/or analyzed during the current study are available from the corresponding author on reasonable request.
